# The role of p53 in cancer drug resistance and targeted chemotherapy

**DOI:** 10.18632/oncotarget.13475

**Published:** 2016-11-19

**Authors:** Karin Hientz, André Mohr, Dipita Bhakta-Guha, Thomas Efferth

**Affiliations:** ^1^ Department of Pharmaceutical Biology, Institute of Pharmacy and Biochemistry, Johannes Gutenberg University, Mainz, Germany; ^2^ School of Chemical and Bio Technology, SASTRA University, Tamil Nadu, India

**Keywords:** cytotoxic chemotherapy, drug resistance, medicinal chemistry, prognostic factors, targeted chemotherapy

## Abstract

Cancer has long been a grievous disease complicated by innumerable players aggravating its cure. Many clinical studies demonstrated the prognostic relevance of the tumor suppressor protein p53 for many human tumor types. Overexpression of mutated p53 with reduced or abolished function is often connected to resistance to standard medications, including cisplatin, alkylating agents (temozolomide), anthracyclines, (doxorubicin), antimetabolites (gemcitabine), antiestrogenes (tamoxifen) and EGFR-inhibitors (cetuximab). Such mutations in the *TP53* gene are often accompanied by changes in the conformation of the p53 protein. Small molecules that restore the wild-type conformation of p53 and, consequently, rebuild its proper function have been identified. These promising agents include PRIMA-1, MIRA-1, and several derivatives of the thiosemicarbazone family. In addition to mutations in p53 itself, p53 activity may be also be impaired due to alterations in p53s regulating proteins such as MDM2. MDM2 functions as primary cellular p53 inhibitor and deregulation of the MDM2/p53-balance has serious consequences. MDM2 alterations often result in its overexpression and therefore promote inhibition of p53 activity. To deal with this problem, a judicious approach is to employ MDM2 inhibitors. Several promising MDM2 inhibitors have been described such as nutlins, benzodiazepinediones or spiro-oxindoles as well as novel compound classes such as xanthone derivatives and trisubstituted aminothiophenes. Furthermore, even naturally derived inhibitor compounds such as a-mangostin, gambogic acid and siladenoserinols have been discovered. In this review, we discuss in detail such small molecules that play a pertinent role in affecting the p53-MDM2 signaling axis and analyze their potential as cancer chemotherapeutics.

## INTRODUCTION

### p53 unfurled

*TP53* (tumor suppressor gene p53) is one of the most well-studied tumor suppressor genes. Because of its pivotal role in protecting from malignancies, p53 is called “guardian of the genome” [1–4]. Its signaling is triggered through myriad cellular events ranging from DNA damage to hypoxia, stress and a plethora of other causes [2, 3, 5–7]. Upon activation, p53 acts as zinc-containing transcription factor [7–11] and regulates downstream genes that are involved in DNA repair, cell cycle arrest or apoptosis [6, 7, 12–15]. Apoptosis is initiated by trans-activating pro-apoptotic proteins such as PUMA (p53 upregulated modulator of apoptosis) [15, 16], FAS (cell surface death receptor) [2, 15], or BAX (Bcl-2-associated X protein) [2, 6, 7, 15–17]. In contrast, cell cycle arrest is induced by p53 via trans-activating genes such as p21 (CDK-inhibitor 1, cyclin dependent kinase) [2, 6, 7, 15] and others [3, 15]. Interestingly, p53 itself is capable of triggering cellular responses (survival or induced cell death) as well. This ability may vary according to the cell type, intensity of stress signal and/or extent of cellular damage [15]. Besides an augmentation of the protein level, the activation of p53 also includes post-translational modifications in the protein itself, which subsequently activates p53-targeted genes [18]. One such post-translational modification is induced by DNA damage. Similar damage leads to activation of kinases like ATM (Ataxia telangiectasia-mutated protein) [3, 4, 17, 18] and Chk2 (Checkpoint kinase 2), which subsequently phosphorylate p53, resulting in p53-dependent cell cycle arrest or apoptosis [18]. In normal cells, expression of p53 is low [7, 13] and its half-life is about 20 min [13]. However, in the case of cellular stress, p53's half-life is extended to several hours, which consequentially results in elevated p53 protein levels in the cell [18]. As cellular gatekeeper [7, 12, 18, 19], a primary role of p53 is to recognize, whether damage is irrevocable and accordingly induce apoptosis [18, 19].

### The involvement of p53 in cancer

It is well known that p53 suppresses tumor formation and renders protection against DNA damage by inducing cell cycle arrest, DNA repair, or apoptosis [2, 6, 7, 20, 21]. However, the p53 pathway is often mutated in cancer [12]. In fact, mutations or deletions in the *TP53* gene are present in nearly 50% of human cancers, and primarily results in impaired tumor suppressor function [22]. Upon loss of p53 functionality, damaged cells may proliferate transferring mutations to the next generation [20]. It is through this mechanism that deregulation of p53 often leads to the formation of tumors [20].

Cancers harboring mut-p53 (mutant p53) are commonly characterized by aggravated metastasis and genomic instability [23, 24]. Several *in vitro* studies have exhibited additional oncogenic functions of mut-p53 in addition to tumor suppression. These functions include promoting invasion, migration, angiogenesis and proliferation [23]. To worsen the matter further, mut-p53 is also responsible for enhanced drug resistance and mitogenic defects [23]. The above functions are just a few of the plethora of characteristics attributed to p53. This suggests the presence of multiple pathways, through which p53 asserts a crucial role in cancer progression that are impacted by mut-p53 [23].

Mutations in p53 may arise due to an anomaly in the position of any amino acid [23]. However, multiple reports indicate preferred sites of mutation: R175, G245, R248, R249, R273, and R282 [23]. Mut-p53 can be broadly classified into structural and DNA-contact mutants. While the former causes unfolding of wild-type p53 (wt p53) protein, the latter changes single amino acids, disabling the binding of p53 to DNA (53). The conglomeration of mutations indicates that the DNA-binding activity of p53 is the main critical function, which is commonly modified in p53 mutants [23, 25]. It is interesting to note that the majority of mutations occur in the core domain of p53, which usually harbors sequence-specific DNA binding activity (residues 102-292) [26]. Consequently, mutations in this site result in the loss of DNA binding.

Muller and Vousden have described four different mechanisms of mut-p53 [23]. These mechanisms stand testimony to the changes in the interaction between mut-p53 and other proteins. Among these proteins, transcription factors play a crucial role. In model 1, mut-p53 interacts with DNA by using mut-p53 binding elements or directly interacts with other parts of the DNA. In model 2 and 3, mut-p53 binds to transcription factors or other proteins and selectively enhances (*e.g*. NF-Y, nuclear factor Y) or inhibits (*e.g*. p63) their functions. Proteins that are not directly associated to the regulation of gene expression (*e.g*. NRD1, nardilysin 1) can also be enhanced or blocked by mut-p53 (model 4). Alteration in DNA-binding ability is yet another important mechanism through which mut-p53 has been observed to function [23]. Thus the manifold contributions of p53 to disease progression makes exploring its role in pathogenesis indispensable.

### MDM2 antagonizes p53

**Figure 1:**
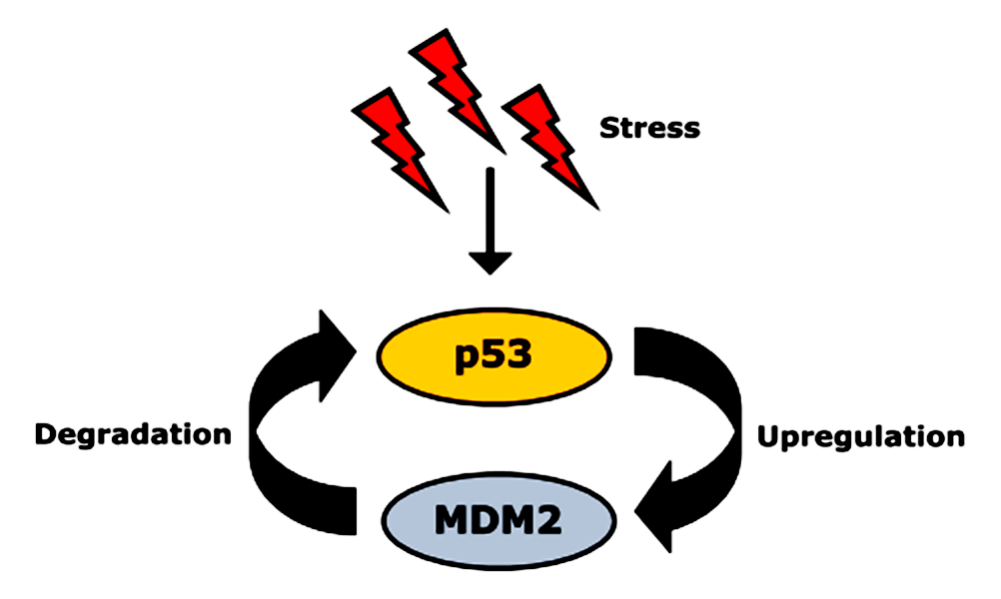
Autoregulatory feedback loop between p53 and its negative regulator, MDM2 After activation due to stress signals, p53 upregulates *MDM2* gene expression and increases MDM2 protein levels. As negative regulator, MDM2 protein then binds to p53 and induces its degradation [22].

Despite the significant role of mut-p53 in cancer pathobiology, the intriguing fact remains that half of all cancer patients carry wt p53 [27]. This indicates the existence of other factors that favor tumor formation and progression. One such factor is the p53-inhibitory protein MDM2 (murine double minute 2). MDM2 is the key physiological regulator of p53 and tightly controls p53 protein levels [12] (Figure 1). It contains a RING (really interesting gene) domain at the C terminus functioning as an E3 ligase along with a p53-binding domain at the N terminus [16]. Wild-type p53 acts as transcription factor, activating MDM2 to target p53 for degradation [7, 9–11, 28]. p53 binds to the MDM2 promoter, thereby increasing MDM2 expression [22]. While on the one hand MDM2 binds to p53 and blocks its N-terminal transactivation domain, it induces p53's degradation via ubiquitin-proteasome machinery on the other hand [12]. After cellular stress such as DNA damage, MDM2's activity decreases leading to p53 stabilization [11, 28–30]. The resulting increase in p53 protein levels leads to an upregulation of MDM2 activity, which in turn causes degradation of p53 [10, 28, 31]. Thus, the cellular levels of p53 and its inhibitor MDM2 are in turn, mutually controlled by negative feedback loop [9–11, 28, 30], wherein MDM2 downregulates p53 and p53 upregulates MDM2. As a result of this regulatory circuit, the nuclear concentrations of both p53 and MDM2 are normally kept at low levels [12]. Therefore, a deregulated MDM2/p53 balance, (*e.g*. by overexpression of MDM2) may have devastating consequences [22].

Such imbalance is often associated with malignant changes in normal cells. Increased MDM2 levels lead to tumorigenesis and correlate with poor clinical prognosis [22]. The overall frequency of *MDM2* gene amplification in human tumors is around 7%. In a study comprising approximately 4000 biopsies obtained from clinical or xenograft tumors across 28 tumor types, the frequency of *MDM2* amplifications varied over a wide range in different types of cancer. The highest frequency of *MDM2* amplification was observed in soft tissue tumors (20%), and osteosarcomas followed second with an incidence of 16% [32]. Several mechanisms have been suggested for MDM2 overexpression. In addition to gene amplification, a specific single nucleotide polymorphism (SNP) in the *MDM2* gene promoter (SNP309) leads to enhanced transcription and increased translation [22, 33]. Therefore, the interaction between MDM2 and p53 suggests an appealing strategy for treatment of cancer and represents a promising therapeutic target [16].

## RESISTANCE TO STANDARD MEDICATION

The relationship between a target and a drug does not stand alone. Any drug-target interaction is plagued with many other factors that pose threats to the holistic therapeutic strategy. One such factor is drug resistance. Resistance towards cytotoxic drugs still presents an enormous problem in the treatment of cancer [34]. Some mechanisms of drug resistance have been recently classified by Marin et al. The authors distinguished between different classes of mechanisms: those involving drug uptake or export, mechanisms concerning metabolic prodrug activation or drug inactivation, changes in molecular targets, as well as mechanisms regarding DNA repair or modifications in the pro- and antiapoptotic balance [151]. Table 1 gives an overview of mechanisms of drug resistance.

**Table 1:** General mechanisms of drug resistance classified in groups [35]

Mechanism of Chemoresistance		Reference
Drug Uptake	Transporters of the superfamily of solute carriers (SLCs) play an important role in the uptake of cytotoxic drugs. If changes occur in the expression of these transporters, tumor cells are less able to take up anticancer drugs leading to the development of resistance.	[36, 37]
Drug Export	Overexpression of members of the superfamily of ATP-binding cassette (ABC) proteins represents another major problem in resistance to chemotherapy. MDR1 (multidrug resistance protein 1, ABCB1 gene) is one of such ABC protein. It acts by bumping out potentially toxic compounds and is therefore also limiting the intracellular concentration of cytotoxic drugs.	[35, 38]
Metabolic Prodrug Activation or Drug Inactivation	Changes affecting the drug metabolism are another reason for resistance. Tumor cells overexpressing detoxifying phase I and phase II enzymes possess an enhanced ability to inactivate cytotoxic drugs. An increased CYP3A4 activity, an enzyme of the cytochrome P450 family, inactivates for example paclitaxel in colorectal cancer cells. Furthermore, even a reduced expression of drug activators led to reduced drug sensitivity. For example carboxylesterases, normally involved in intracellular activation of irinotecan, are reduced in cancer cells with enhanced resistance to irinotecan.	[35, 39], [40]
Changes in Molecular Targets	Changes in molecular targets and defective signaling pathways are altering the sensitivity of tumor cells to anticancer drugs. For example, the mechanism of action of anthracyclines is based on their ability to interact with DNA topoisomerases. Mutations in the *TOP1* gene, encoding topoisomerase 1 led to a reduced ability of anthracyclines to interact with their target.	[35]
DNA Repair	The enhanced ability of tumors cells to repair drug-induced DNA damages leads to resistance. Nucleotide excision repair is one major DNA mechanism, resulting from the use of alkylating agents. Furthermore, mismatch repair (MMR) is involved in the correction of erroneously matched nucleotides. The loss of MMR activity causes genetic instability with enhanced resistance to a large variety of anticancer drugs.	[35, 41, 42]
Modifications in the Pro- and Antiapoptotic Balance	Modifications of key factors of apoptosis such as p53 play a major role in resistance. Also *BAX* function is often impaired due to mutations in the BAX gene, leading to the synthesis of truncated proteins and the development of oxaliplatin-resistant cells. Furthermore, also the upregulation and abnormal expression of antiapoptotic factors such as Bcl-2 (B-cell lymphoma protein 2) or Bcl-XL (B-cell lymphoma protein extra-large) are connected to enhanced resistance.	[35, 43]

Mutations in the p53 gene are modifications in the proapoptotic balance causing drug resistance (Table 2). Alterations in p53 frequently occur in tumors and result in loss of p53 function [35]. The correlation between p53 mutation status and sensitivity to cytotoxic drugs has been confirmed by a large study conducted by the National Cancer Institute, USA, where 60 cell lines and more than 100 anticancer drugs were examined [36]. However, the way in which p53 influences drug resistance depends on several different parameters including the mode of action of the drug, genetic alterations during carcinogenesis, and the type of cancer [35]. A comprehensive overview of mechanisms of resistance development against all cytotoxic drugs is beyond the scope of a single review article. We therefore have selected several chemotherapeutic agents and described their mechanisms of drug resistance in Table 2.

**Table 2:** Resistance towards standard medications in mut-p53 harboring cancer cells

Agent	Agent Class	Mechanism of Drug Action	Possible mechanism of Resistance in p53 mutant cells	Reference
Cisplatin	Platinum-based complex	Inhibition of DNA replication by DNA cross-linking after Cl-elimination.	Mutant p53 upregulates Nrf2 (nuclear factor erythroid 2 –related factor 2, a transcription factor coding for detoxification enzymes and conferring resistance to anticancer drugs) in non-small cell lung cancer by increased binding to the Nrf2 promoter supported by an activation of the NF-κB signaling pathway leading to additional enhancement of Nrf2 expression. Furthermore, loss of DNA mismatch repair favors cisplatin resistance in p53 mutant colon carcinoma cells.	[46–50]
Temozolomide	Alkylating agent	DNA damage and inhibition of cell division by inserting alkyl groups in the DNA.	In temozolomide-resistant glioma cells, a correlation between mutant TP53 and MGMT (O6-methylguanine DNA-methyl-transferase) was observed. While temozolomide kills cells by alkylating O6-guanine, MGMT in turn repairs alkylation. Therefore drug resistance may be caused by MGMT up-regulation.	[15, 51–56]
Doxorubicin	Anthracycline	Intercalation into DNA and inhibition of DNA-topoisomerase II leading to DNA damage and apoptosis.	TP53 mutations affecting or disrupting the zinc atom chelating, L2/L3 DNA binding domains of the p53 protein are linked to primary resistance to doxorubicin therapy in breast cancer. Furthermore polymorphism in codon 72 (Arg/Pro) of p53 affects cellular sensitivity to anticancer drugs such as doxorubicin through inhibition of p73, a protein related to p53.	[57–62]
Gemcitabine	Antimetabolite	Interference of normal metabolism due to the masquerade of antimetabolites as natural metabolic element. This inhibits normal cell development and cell division.	Gemcitabine treatment stabilizes mutant p53 in the nuclei and induces the expression of mutant p53 target genes CdK1 (cyclin-dependent kinase 1) and CCNB1 (G2/mitotic-specific cyclin-B1), which are both involved in mitosis and therefore cell proliferation, leading to gemcitabine resistance in pancreatic cancer cells.	[63–65]
Tamoxifen	SERM (selective estrogen receptor modulator)	Suppression of ER (estrogen receptor)- mediated gene expression and cell proliferation due to antagonizing ERs. Especially, tamoxifen can exert both agonistic and antagonistic activity depending on the target tissue and can therefore be considered as SERM.	Expression of ER and p53 is mutually regulated through a feedback loop. While ER upregulates p53 expression by protein stabilization and transcriptional regulation, p53 upregulates ER again. That may explain why mutations in p53 would inhibit ER expression, decreasing the effects of tamoxifen in breast cancer and leading to drug resistance.	[66–71]
Cetuximab	EGFR (epidermal growth factor receptor) -inhibitor	Monoclonal antibodies block epidermal growth factor receptor (EGFR), inhibiting signal transduction and therefore leading to reduced tumor growth.	Mutant p53 influences ERK (extracellular-signal regulated kinases) pathway and ERK-mediated transcription of Egr-1 (early growth response-1), which in turn increases the secretion of EGFR ligands, causing stimulation of EGFR signaling and therefore making EGFR-inhibitor treatment ineffective.	[72–74]

## AGENTS RESTORING P53 WILD-TYPE CONFORMATION

**Figure 2:**
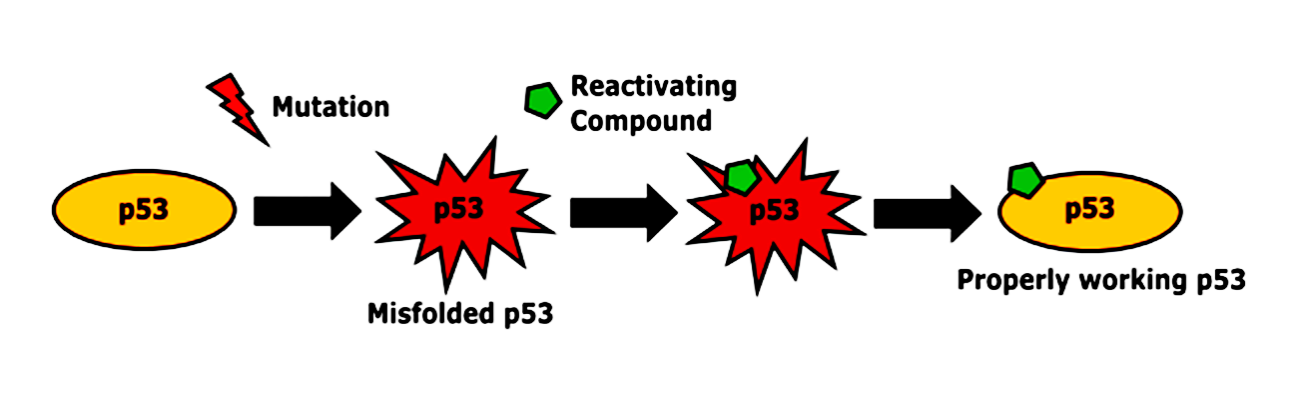
Restoration of p53 conformation by reactivating substances In cancer cells, the p53 protein is often inactivated by mutations. By binding misfolded and inactivated p53, reactivating compounds can restore its active form and tumor suppressor function [67].

Missense mutations in *TP53* often result in the accumulation of mutant and misfolded proteins in the nucleus [66]. Re-folding of this mutated and accumulated p53 leads to restoration and activation of defective proteins, resulting in high levels of active p53, which then induces apoptosis [67]. Therefore, small molecules capable of restoring p53 function, pose an attractive new class of anticancer drugs (Figure 2).

In the past years, several mut-p53-reactivating compounds have been described. An overview is given in Table 3.

**Table 3:** Overview of agents reactivating mut-p53

Agent	Chemical Structure	Agent Class	Mechanism of Action	Reference
MIRA-1	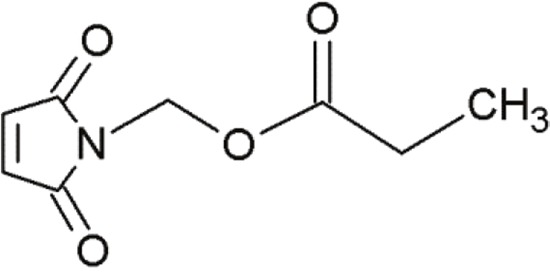	Maleimide analogues	Restoring native conformation by alkylation of thiol groups in mut-p53	[68]
PRIMA-1	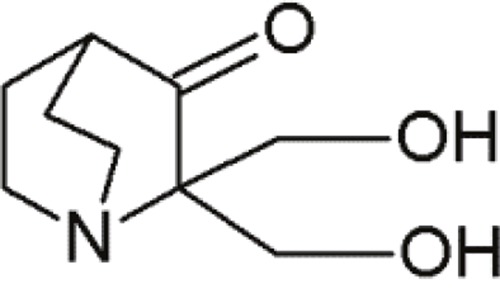	Quinuclidines	Restoring native conformation by alkylation of thiol groups in mut-p53	[69]
PRIMA -1Met (APR-246)	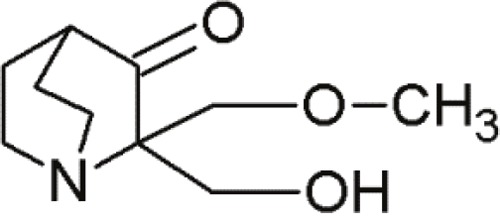	Quinuclidines	Restoring native conformation by alkylation of thiol groups in mut-p53	[69]
NSC319725	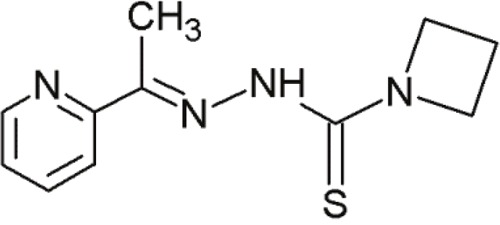	Thiosemicarbazone family	Serving as source of zinc to allow mut-p53 refolding into its wild-type conformation	[70]
NSC319726	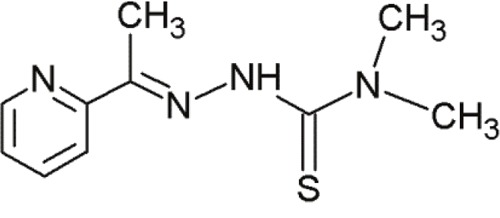	Thiosemicarbazone family	Serving as source of zinc to allow mut-p53 refolding into its wild-type conformation	[70]
NSC328784	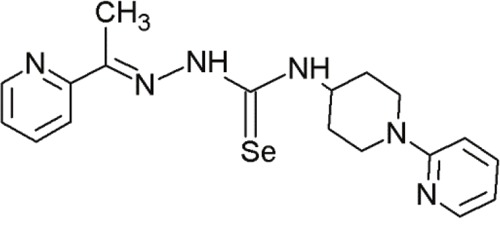	Thiosemicarbazone family	Serving as source of zinc to allow mut-p53 refolding into its wild-type conformation	[70]
PhiKan083	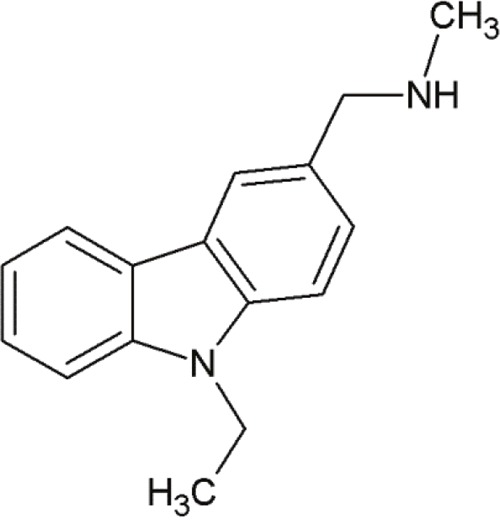	Carbazole-derivative	Binding at Y220C mutation site on p53 and slowing down its thermal denaturation rate	[71]

### Thiosemicarbazones

A novel class of compounds that act as re-activators of mut-p53 has been investigated by Yu and co-workers [8]. Using the compound library of the National Cancer Institute (NCI), USA; they identified three compounds (NSC319725, NSC319726, NSC328784) showing promising inhibitory effects in mut-p53 cell lines [8]. Comparing cell lines with mut-p53 and wt-p53, significantly lower IC_50_ values have been found in cells with the mutated status. NSC319726 exhibited particularly low IC_50_ values in cells with a p53 mutation in the 175 allele. Furthermore, the compound was remarkably non-toxic to human fibroblasts (with wt-p53). The authors observed a two-fold higher induction of apoptosis in the p53^R175^ mutant than in other mutant cell lines. Upon silencing the expression of mutant p53^R175^ protein, the sensitivity to these compounds was strongly reduced. This led to the conclusion that the activity of NSC319726 was dependent on p53^R175^ mutant protein [8].

Wt-p53 includes one zinc ion as an important cofactor, which is coordinated to the side chains of three cysteine residues and one histidine residue in the DNA-binding domain. The metal ion stabilizes the second and third loop within the DNA-binding domain and is essential for its correct function. In p53 mutants on the other hand, zinc is recurrently missing [72]. Mutations in any of the zinc-coordinating residues result in the inability of p53 to bind to zinc. Although the R175 mutant is not directly involved in zinc binding, a histidine residue at this location is capable of inducing structural distortions in the protein, thereby preventing binding of zinc [70].

Generally, metal ion chelation changes p53 conformation [73, 74]. Therefore, NSC319726 treatment induces a wild-type-like conformational change in the p53-mutant protein, because of its metal-ion chelating properties. The R175 mutant p53 fails to bind zinc, but upon treatment with NSC319726 and zinc chloride at low concentrations, zinc binding was detected [70]. Therefore, NSC319726 may serve as source of zinc to allow the p53^R175^ mutant to refold to its wild-type conformation [70].

NSC319726 may be a promising compound for three reasons: firstly, because p53^R175^ mutant reactivation has been observed *in vivo* at doses that are non-toxic to human tissues harboring wt-p53. Secondly, the compound exhibited a wide therapeutic window. Thirdly, the target, mut-p53 protein, is frequently found in tumor cells. According to the International Agency for Research on Cancer (IARC) *TP53* database, the 175 mutant is the third most commonly found missense mutation in *TP53* [70, 75].

### PhiKan083

Rauf et al. presented another small molecule mut-p53 reactivator, PhiKan083 [72]. It is a carbazole-derivative, which binds at the Y220C mutation site on p53 and slows down its thermal denaturation rate [71]. Y220C is one of the most frequent mutations in cancer [76, 77] and is located outside the DNA-binding surface of p53. In this mutation, the replacement of a tyrosine by a cysteine leads to decreased thermodynamic stability [72]. Although the mutation does not change the overall structure of the protein [78], several electrostatic interactions are lost. More precisely, the S7/S8 loop of the protein is destabilized, because Tyr220 cannot build electrostatic interactions with the residues Val147, Pro151, Pro153 and Pro223. This leads to the displacement of the loop from its original position [72].

A drugable cavity is created on the surface at the mutation site, which is far from the functional region of p53. PhiKan083 occupies this cavity and is involved in electrostatic interactions with Pro155, Glu221 and Thr230 as well as hydrogen bonds with Leu145 and Asp228. These interactions stabilize the S7/S8 loop and prevent its displacement. As a result of loop stabilization, PhiKan083 raises the melting temperature of Y220C mutant p53 and consequently decreases thermal denaturation [79].

### PRIMA-1 and PRIMA-1Met

Another compound that represents a breakthrough in the reactivation of p53 is p53-Reactivation and Induction of Massive Apoptosis-1 (PRIMA-1), which was identified by Wiman and colleagues [79]. This quinuclidine compound rescued the DNA-binding activity *in vitro* in numerous mut-p53 protein species [66]. Its methylated analogue PRIMA-1^Met^ (also known as APR246) showed even greater potential [67].

Treatment with quinuclidines upregulated p53 target genes such as *BAX, PUMA* and *NOXA* [79]. Furthermore, PRIMA-1 and APR-246 induced activation of caspases -2, -3 and -9 [8]. Under physiological conditions, PRIMA-1 and APR-246 are rapidly converted into a reactive intermediate compound, MQ (methylene quinuclidinone). MQ acts as Michael acceptor and covalently binds to the core domain of p53 [80]. Conversion of PRIMA-1 and APR-246 to MQ renders them biologically active. As expected, derivatives that cannot be transformed into this reactive form are biologically inactive. Therefore, PRIMA-1 and APR-246 can be considered as prodrugs, which need to be activated *in vivo* [79]. However, due to its limited stability at physiological conditions, MQ itself cannot be used as a mut-p53-targeting compound and thus necessitates further improvement [80].

Due to incorrect folding, mut-p53 proteins may expose cysteine residues, which are hidden in the core domain of wt-p53 [79]. This can lead to the formation of inter- and intramolecular disulfide bonds, locking mut-p53 in an inactive conformation and causing protein aggregation. Thiol modification by reactive compounds such as MQ prevented the formation of such disulfide bonds and thus promoted correct folding and restoration of the wild-type function [8].

However, it is not clear which of the 10 cysteine residues in the p53 core domain are modified by MQ. Cys182, Cys229, Cys242 and Cys277 are all exposed on the surface of the core domain, and are potential targets for modifications [64, 81]. According to Lambert et al. mut-p53 is more amenable to this type of covalent modification than the wild-type version [80]. Interestingly, unfolded wt-p53 proteins are also modified by MQ. The degree of binding is correlated to the extent of unfolding [80].

In addition, a further benefit of PRIMA-1 is represented by its large therapeutic window [79]. Although normal cells seem to be more resistant at therapeutic doses, PRIMA-1 and APR-246 may target other proteins in addition to p53 [8]. How does MQ then achieve target specificity? One plausible explanation is that the structural aspect of a specific cysteine might dictate selectivity and cater to the modification of only a limited number of proteins. Furthermore, the active product, MQ, is generated from a prodrug, only after cellular uptake [8]. This may prevent extensive modification of extracellular proteins.

Several explanations have been suggested, as to how alkylation of thiol groups in mut-p53 could restore native conformation and function: (i) prevention of disulfide bond formation within the core domain of p53, (ii) alkylation-mediated escalation in the protein fraction capable of binding to DNA, (iii) formation of adducts in the core domain that may allow effective DNA-binding and consequent transactivation of p53 target genes, and (iv) PRIMA-1 adducts that create additional hydrophobic interactions with amino acids in the core *via* hydrogen bonding, thereby promoting accurate folding of the core [80].

Li et al. proposed new insights into PRIMA-1^Met^ functionality [82]. PRIMA-1^Met^ is the methylated analogue of PRIMA-1, in which one of the two hydroxyl-groups is replaced by a methoxy-group [82]. PRIMA-1^Met^ limited the growth of colorectal cancer (CRC) cells irrespective of p53 status, although robust apoptosis was induced only in mut-p53 cells [82]. Upregulation of the pro-apoptotic protein NOXA was essential for PRIMA-1^Met^-mediated activity. It led to apoptosis associated with cleavage of PARP [Poly (ADP-ribose)-Polymerase, involved in DNA repair and programmed cell death] [82]. Contradictory to several previous studies, Li et al. reported that CRC cell lines with wt-p53 or p53-null cells also responded to PRIMA-1^Met^ treatment [82]. Bykov and Wiman have put forward one credible mechanism accounting, at least in part, for the effects of the drug in p53-null cancer cells [69]. APR-246, on conversion into MQ, modifies TrxR1 (thioredoxin reductase 1) into an oxidase. The latter induces reactive oxygen species (ROS), which in turn contribute to APR-246-induced cell death [69]. However, in CRC cells with wt-p53 or p53-null cells, PRIMA-1^Met^ mainly induces cytostasis, whereas the induction of apoptosis appears specifically in mut-p53 cells [82]. This study by Li et al. opens up avenues to attain greater efficacy in the use of PRIMA-1^Met^ against cancer. However, a deeper understanding of the molecular mechanism is still required [82].

### MIRA-1

Another mut-p53 function-restoring small molecule is MIRA-1 (mut-p53-dependent induction of rapid apoptosis) [67]. Although structurally MIRA analogues are distinct from PRIMA-1, the molecular mechanism involving reactivation of mut-p53 by MQ is quite similar [68]. MIRA-1 contains a maleimide group with 3-4 double bonds, which forms chemical bonds with thiol and amino groups. Thus, alkylation of cysteine and/or lysine residues of p53 by MIRA-1 stabilizes native folding of the protein. The alkylation status, however, depends on the accessibility of thiol groups. As a consequence, mutant and unfolded proteins are more effectively modified by MIRA-1 than is the correctly folded wild-type [67, 83].

Although MIRA-1 and PRIMA-1 both act *via* modification of thiol groups, they differ considerably in kinetics of cell death induction. MIRA-1 induces cell death much faster than PRIMA-1 does (6-12 h *versus* 24-48 h). This could be attributed to the variable dynamics of cellular uptake or degradation as well as differences in their modes of action [67, 84]. MIRA-1 induces cell killing in a mut-p53-dependent manner with a much higher potency than PRIMA-1, and MIRA-1-induced cell death involves DNA-fragmentation as well as caspase activation [67].

The maleimide groups of MIRA analogues react with thiol and amino groups of the protein through nucleophilic addition [67]. Presence of several double bonds within the maleimide group is critical for its activity. MIRA-1 acts by shifting the equilibrium of p53 towards the native conformation, which leads to restoration of p53-mediated transactivation of target genes such as *p21, MDM2* (murine double minute 2) and *PUMA* as well as induction of p53-dependent apoptosis [67].

MIRA-1 acts on multiple inter-connected pathways to induce apoptosis. In a recent study, Saha et al. evaluated the anti-myeloma activity of MIRA-1, both *in vivo* and *in vitro* [85]. The authors showed that the p53 status is not a decisive factor for induction of apoptosis by MIRA-1 in multiple myeloma (MM) cells. The group used wt-, mut- and silenced p53 MM cells and observed that MIRA-1 treatment resulted in the induction of multiple signaling pathways implicated in apoptosis of MM cells [85]. Firstly, MIRA-1 treatment induced ER-signaling [85–87] by triggering activation of PERK (protein kinase R-like endoplasmic reticulum kinase), one of the ER stress sensors. PERK as well as eIF2-α (eukaryotic initiation factor 2) are phosphorylated and subsequently several chaperone proteins are activated (calnexin, protein disulfide isomerase (PDI) and binding immunoglobulin protein (BiP)) [85]. Consistent with their findings, the activation of pro-apoptotic PUMA and BAX as well as the repression of anti-apoptotic proteins like Mcl-1 and c-Myc have been shown to be linked to ER stress signaling pathways. Thus, they concluded that contrary to previous assumptions, MIRA-1 induces p53-independent apoptosis in MM cells primarily because of a change in the balance between pro- and anti-apoptotic proteins [85].

However, further studies are required to fully understand the modulatory mechanisms of MIRA-1-associated intervention strategies in intracellular cross-talks leading to apoptosis. Simultaneously, the quest to identify prominent signaling molecules within those pathways becomes pertinent.

## AGENTS INTERRUPTING THE MDM2-P53-INTERACTION

Despite the fact that nuclear levels of both p53 and MDM2 are normally kept at low levels due to a regulatory circuit, a deregulated MDM2/p53 balance, (*e.g*. by overexpression of MDM2) diminishes the tumor suppressive functions of p53 [12, 22]. Owing to this antagonizing effect of MDM2 on p53, small molecules have been developed that mimic p53-binding residues [37]. High-resolution crystal structures of MDM2 and p53 indicate that their interaction is mediated by a well-defined hydrophobic surface pocket and three hydrophobic key residues: Phe19, Trp23 and Leu26 [88]. The compact binding pocket in MDM2 enables scientists to design new small molecules capable of blocking the MDM2-p53 interaction. With this interaction blocked, p53 is no longer controlled by MDM2 and is reactivated in tumor cells harboring wt-p53 [38].

However, MDM2 is not the only negative regulator of p53 and therefore the other ones have to be kept in mind. P53 is cross-linked in a complex network with several other actors. For example, it is also regulated by the proteins SirT1 (Sirtuin 1) [160] or Wip1 (wt-p53 induced phosphatase/PPM1D) [162].

Sirtuin 1 is a NAD+-dependent histone deacetylase, which also deacetylates non-histone proteins involved in cell growth, apoptosis, tumorigenesis and cell senescence [160]. P53 becomes acetylated after DNA damage leading to increased transcriptional activity. SirT1 interacts with p53 and attenuates p53-mediated functions through deacetylation of p53 at its C-terminal residue (Lys382). Therefore, overexpression of SirT1 leads to the repression of normal p53-dependent response to DNA-damage or oxidative stress such as cell cycle arrest and apoptosis [160, 161].

Furthermore, the interaction between p53 and its other negative regulator Wip1 is controlled through an autoregulatory feedback loop, similar to MDM2. Wip1 is induced by genotoxic stress. It efficiently inhibited the p53 pathway by dephosphorylation of p53 at its transactivating domain Ser15 as well as by dephosphorylation of MDM2. Therefore, Wip1 promoted recovery from the G2 checkpoint and contributed to the termination of DNA damage response [162, 163, 164]. These facts affirm the necessity to decode the entire p53 interaction network as it would open new fields of research of novel chemotherapeutic agents.

Sriraman et al. tested, whether the simultaneous inhibition of both p53-antagonists, MDM2 and Wip1, induce p53 activation more potently than single inhibitors. Indeed, the inhibition of Wip1 fortified the effect of MDM2 antagonists alone on p53 activation [162]. Similar results were obtained by others [163, 164]. These findings emphasize the therapeutic potential of negative regulators of p53 such as MDM2, as well as other negative regulators and opens new possibilities of a multitarget chemotherapy in tumors harboring wt-p53.

Numerous MDM2 inhibitors have been developed during the past few years. An overview of some potent ones is presented in Table 4.

**Table 4:** Overview of important and promising MDM2 inhibitors

Agent	Chemical Structure	Agent Class	Mechanism of Action	Reference
Nutlin-3	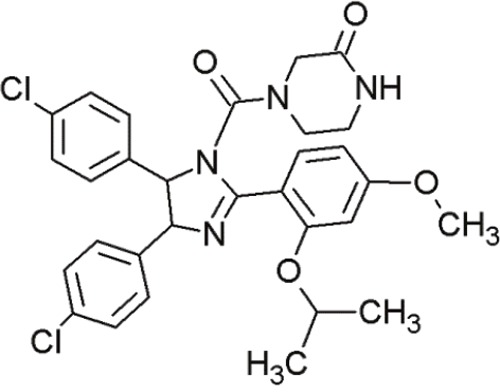	Cis-imidazolines	Blocking the p53-binding pocket on MDM2 by mimicking p53	[83]
Nutlin-3a	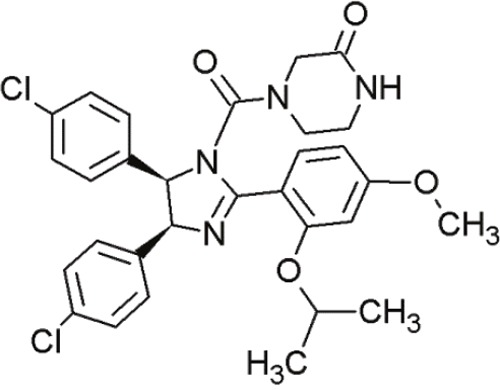	Cis-imidazolines	Blocking the p53-binding pocket on MDM2 by mimicking p53	[83]
RG7112(RO 5045337)	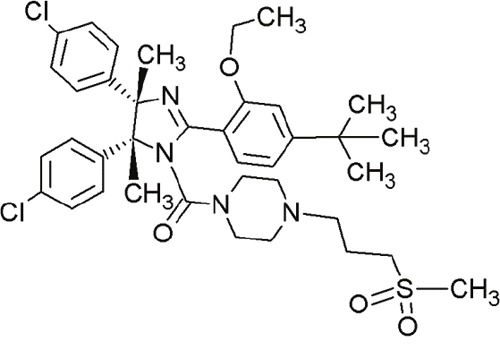	Cis-imidazolines	Blocking the p53-binding pocket on MDM2 by mimicking p53	[89]
RG7388 (RO 5503781)	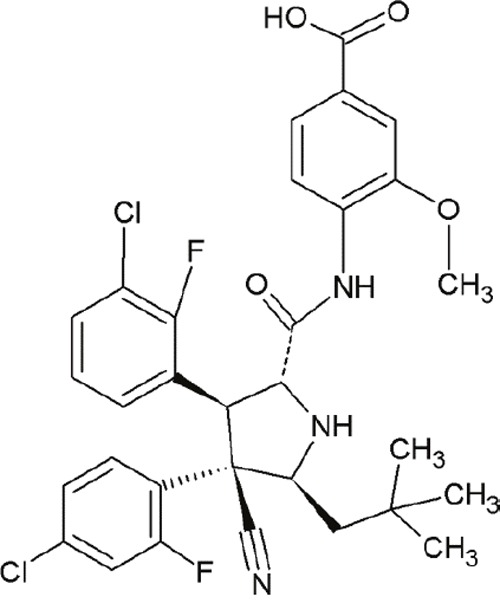	Trans- pyrrolidine derivatives	Blocking the p53-binding pocket on MDM2 by mimicking p53	[90]
MI-219	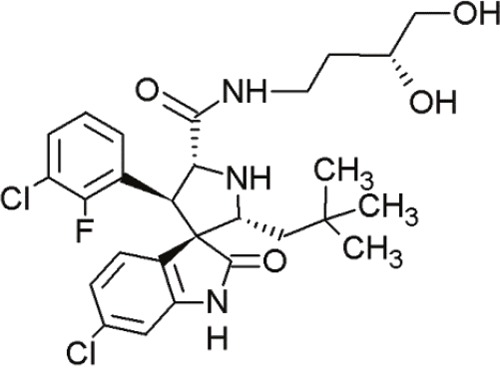	Spiro-oxindoles (MI-series)	Blocking the p53-binding pocket on MDM2 by mimicking p53	[91]
MI-888	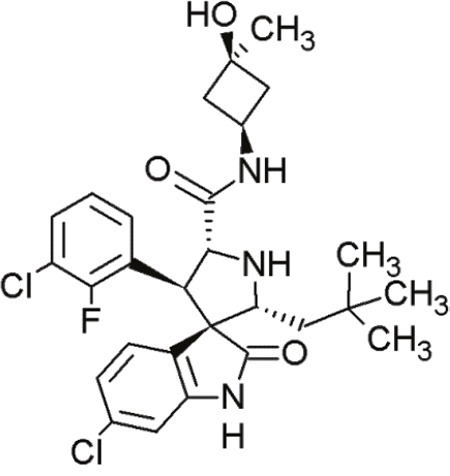	Spiro-oxindoles (MI-series)	Blocking the p53-binding pocket on MDM2 by mimicking p53	[92]
MI-77301(SAR405838)	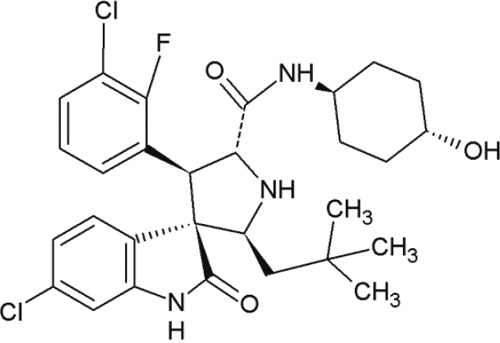	Spiro-oxindoles (MI-series)	Blocking the p53-binding pocket on MDM2 by mimicking p53	[93]
RITA	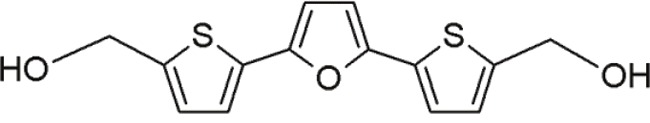	Furan-derivative	Binds the N-terminus of p53 and induces a conformational change which prevents its interaction with MDM2	[94]

### MDM2 inhibitors

#### Nutlins

Cis-imidazolines, also referred to as nutlins, are promising non-peptide small molecules that are well characterized MDM2 inhibitors [95]. They were the first selective small molecules that inhibited the wt-p53-MDM2 interaction [92, 96, 97].

Crystal structure studies demonstrated that cis-imidazolines bind to the p53-binding site of MDM2 by mimicking the interaction of critical amino acid residues [83]. Owing to their hydrophobic nature, Trp23, Phe19 and Leu26 fit excellently into the deep hydrophobic pocket of MDM2 [12].

P53's binding pocket on MDM2 is sterically inhibited by nutlin binding, thus inducing p53 accumulation and restoration of its transcriptional activity followed by apoptosis in MDM2-overexpressing tumor cells [98]. The compounds induce stabilization of p53, induction of p21 target genes, cell cycle arrest as well as apoptosis [99].

Three compounds (nutlin-1, -2 and -3) exhibited suitable IC_50_ values to block the p53-MDM2 interaction [83] (Table 4, Table 5). They possess the same core structure and exhibit only slight variations in their functional groups [95]. These nutlins harbored sufficient cell permeability and elicited dose-dependent accumulation of wt-p53. However, none of them induced cell cycle arrest or upregulated p53 downstream gene targets in mut- or p53-null tumor cells [92]. Hence, it is proposed that only wt-p53 cell lines are sensitive to these compounds. The active enantiomer of nutlin-3a, induced apoptosis in osteosarcoma cells at much lower doses, whereas its inactive enantiomer was ineffective even at higher concentrations [83].

**Table 5:** First nutlin derivatives

Agent	Chemical Structure	Agent Class	Mechanism of Action	Reference
Nutlin-1	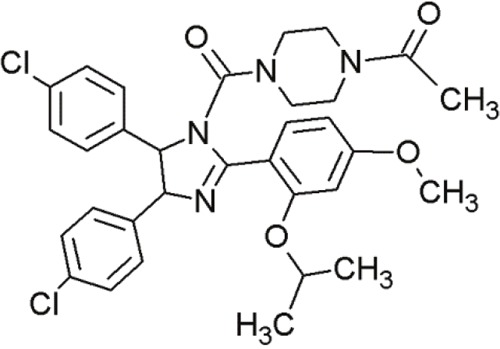	Cis-imidazolines	Blocking the p53-binding pocket on MDM2 by mimicking p53	[83]
Nutlin-2	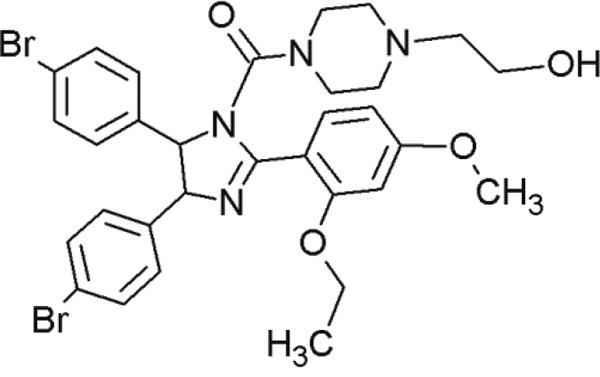	Cis-imidazolines	Blocking the p53-binding pocket on MDM2 by mimicking p53	[83]

Nutlin-3 possessed activity against a broad panel of cancer cells harboring wt-p53 *in vitro* and *in vivo* [96]. It activated p53 transcriptional activity in osteosarcoma, retinoblastoma, lymphoblastic leukemia, colon and breast cancer cell lines [96]. In combination with chemoradiotherapy, the compound showed synergistic activity against prostate [100] and lung cancers [101, 102] as well as lymphocytic leukemia [103] and neuroblastoma [104]. Furthermore, nutlin-3 showed normal cell protective efficacy against the detrimental effects of chemotherapy by inducing cell cycle arrest [105] due to inhibition of BAX and BAK (Bcl2-antagonist/killer) [106].

However, the activity of nutlins strongly suffer from p53 downstream aberrations, such as MDMX overexpression [92]. Overexpressed MDMX (murine double minute x) limits the effectiveness of nutlin-based chemotherapy [92]. Since nutlins do not bind MDMX, cis-imidazolines reduce MDMX protein levels in wt-p53 cancer cells by facilitating MDM2-dependent degradation [107]. In cancer cell lines resistant to cis-imidazolines, a combination therapy with doxorubicin may be effective to overcome resistance. The DNA-damaging anticancer drug doxorubicin effectively depleted MDMX levels and therefore revealed synergistic effects in combination with nutlins [83, 107].

Synergistic effects of nutlin-3 with cytostatic drugs have been reported. Deben et al. investigated the benefits combination of nutlin-3 with cisplatin in sequential treatments (cisplatin followed by nutlin-3) [108]. They used a series of NSCLC cell lines with differential p53 status. Although nutlin-3 showed good efficacy even as single agent, the authors hypothesized that the anti-tumor effect might be enhanced, if given in combination with DNA-damaging agents [108]. Cisplatin and nutlin-3's sequential treatment resulted in a synergistic cytotoxic response in wt-p53 cell lines [108]. A significant increase in p53's targets could be observed. Augmented protein levels of MDM2, p21, PUMA and BAX led to cell cycle arrest at G2/M followed by apoptosis [108]. The group also illustrated that combinatorial therapy of small molecules with DNA-damaging agents (cisplatin) resulted in a synergistic cytotoxic response [108].Unravelling the full importance of nutlin-based combination treatments demands further evaluation.

Another study by Wang and co-workers in 2012 investigated the efficacy of nutlin-3a (Table 4) treatment in osteosarcoma cell lines both *in vivo* and *in vitro* [109]. Employing three osteosarcoma cell lines [U-2 OS (wt p53), SaOS2 (null p53), MG63 (mut-p53)], the authors deciphered that activation of the p53 pathway due to the disruption of p53-MDM2-interaction by nutlin-3a depends on the presence of wt-p53 [109]. Nutlin-3a stabilized p53 and led to dose-dependent anti-proliferative and cytotoxic effects, inducing cell cycle arrest at G1 phase and apoptosis both *in vivo* and *in vitro* [109]. Significant apoptosis and increased G1 phase fractions were detected, if treated with 10 µM nutlin-3a. Further treatment with nutlin-3a significantly upregulated p53 and p21 levels in osteosarcoma cells [109]. In a quest to investigate, whether nutlin-3a suppresses growth of xenograft tumors in nude mice, Wang et al. showed that it was well tolerated at a daily dosage of 25 mg/kg administered intraperitoneally for 14 days. This treatment regimen resulted in 85% inhibition of tumor growth [109].

Tonsing-Carter et al. evaluated the combination treatment for triple negative breast cancers (TNBC) [110]. Due to its aggressive nature (lacking hormone receptors) [111, 112], the development of efficacious therapies for TNBCs is extremely challenging. Tonsing-Carter et al. combined a platinum-based regimen with nutlin-3a. Single and combination treatments of nutlin-3a and carboplatin were tested on a panel of TNBC cell lines with mut-p53 [110]. Due to the inhibition of protein-protein interactions between MDM2 and several of its binding partners including p53, p73α (another tumor suppressor involved in cell cycle regulation and induction of apoptosis with structural resemblance to p53), transcription factor E2F1 (involved in cell cycle and action of tumor suppressor proteins) and Hif-1α (hypoxia inducible factor 1α) [113–115], the use of nutlin-3a led to the activation of several pathways associated with anti-cancer effects [110]. The effect of nutlin-3a was especially beneficial, if it was administered in combination with DNA-damaging drugs. Platinum agents, such as carboplatin, form DNA-platinum adducts leading to double-strand DNA breaks and cell death [116]. *In vitro* studies showed strong synergy between carboplatin and nutlin-3a [110]. In TNBC cells with mut-p53 background, increased cell death was detected, if administered in combination, as well as decreased IC_50_ values for both drugs. Cleaved PARP was detectable in 100% of tumors following combination treatment, whereas it was only detectable in 50 - 66% of tumors treated with carboplatin or nutlin-3a alone [110]. Further inhibition of MDM2 by nutlin-3 increased the pool of available p37α, which in turn activated pro-apoptotic gene expression and finally promoted apoptosis [110]. Combinatorial treatment significantly inhibited tumor growth with no serious physical complaints such as dehydration, diarrhea or bleedings within the gastrointestinal tract [110]. Besides primary tumor growth, even metastatic foci in the lung decreased in size and number relative to single-agent therapy. This was due to an efficient delivery of nutlin-3a to the lung, as suggested by the pharmacokinetic data [110]. Owing to these promising results, combinatorial therapy of nutlin-3a/carboplatin deserve further development as new clinical therapies.

Tovar et al. described a more potent inhibitor of the p53-MDM2 interaction than the original nutlins [89]. RG7112 (Table 4) is an advanced derivative of the class of cis-imidazolines [90]. It displaced p53 peptides from the surface of MDM2 with a 4-fold higher potency than nutlin-3a [96]. Furthermore, RG7112 showed improved binding due to faster on-rate and slower off-rate, and also exhibited improved pharmacological properties [89]. In crystal structure studies, RG7112 was found to bind to MDM2 at the same region as the other nutlins [89]. The two chlorophenyl groups of RG7112 project into the Leu26 and Trp23 pockets and the ethoxy group of the third benzene ring bound into the Phe19 cave of MDM2 [90]. However, compared to the other compounds of the nutlin-group, RG7112 possessed a 4,5-dimethyl substitution at the imidazole ring. This modification caused more structural rigidity, blocking its metabolic conversion in to an inactive imidazole [89].

In addition to these studies, Ding et al. investigated a novel nutlin, RG7388 (RO5503781) (Table 4), which induced p53-stabilization in a dose-dependent manner, as well as cell cycle arrest and apoptosis [90]. Although the mode of action is similar, RG7388 achieved a significantly higher *in vivo* efficacy against human osteosarcoma xenografts tumors than RG7112. Even at lower doses, the efficacy of RG7388 was much higher than RG7112. At 50 mg/kg body weight, RG7112 achieved 74% tumor growth inhibition, whereas RG7388 obtained 84% growth inhibition at much lower doses (12.5 mg/kg) [90]. In conclusion, RG7388 was more potent than RG7112.

### Benzodiazepinediones

1,4-benzodiazepine-2,5-diones (BDPs) are another class of small molecule inhibitors interacting at the p53-MDM2 site [92, 117] (Table 6). Grasberger and co-workers (2005) first reported 1,4-benzodiazepine-2,5-diones as inhibitors of p53-MDM2 interaction [118]. Compound 21 discovered by high-throughput screening of over 338000 compounds achieved a promising IC_50_ value [119]. Modifications of this scaffold led to significant increases in potency. Due to the introduction of a 4-chloro substitution on the phenyl rings of the scaffold, compound 23 exhibited much lower IC_50_ values [120]. An MDM2-23 co-crystal structure demonstrated the interaction between BDPs and MDM2. The three phenyl rings of compound 23 bound to MDM2 by mimicking the conformation of the p53 triad motif [121].

**Table 6:** Overview of some benzodiazepinediones undergoing first steps of investigation

Agent	Chemical Structure	Agent Class	Mechanism of Action	Reference
Compound 23	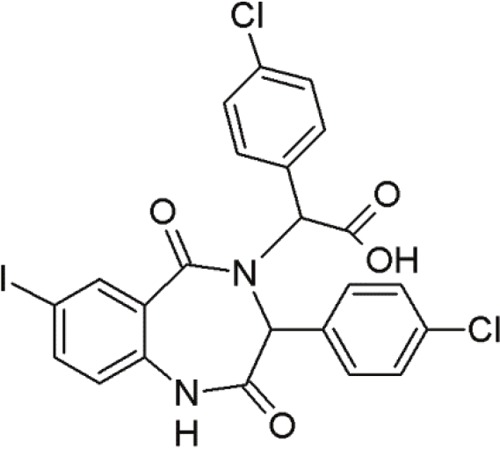	Benzo-diazepine-diones (BDPs)	Blocking the p53-binding pocket on MDM2 by mimicking p53	[83]
Compound 27	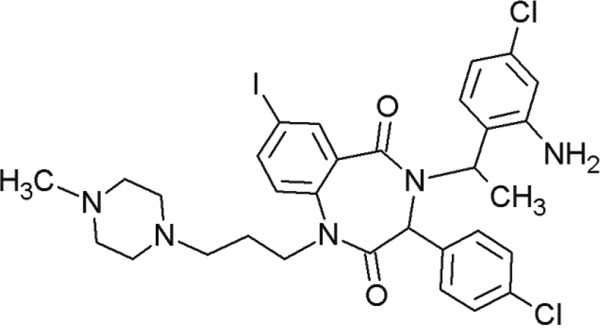	Benzo-diazepine-diones (BDPs)	Blocking the p53-binding pocket on MDM2 by mimicking p53	[83]
8i	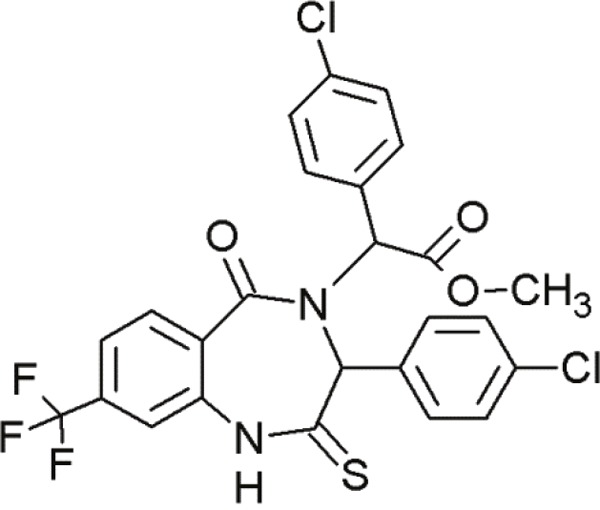	Benzo-diazepine-diones (BDPs)	Blocking the p53-binding pocket on MDM2 by mimicking p53	[117]
8n	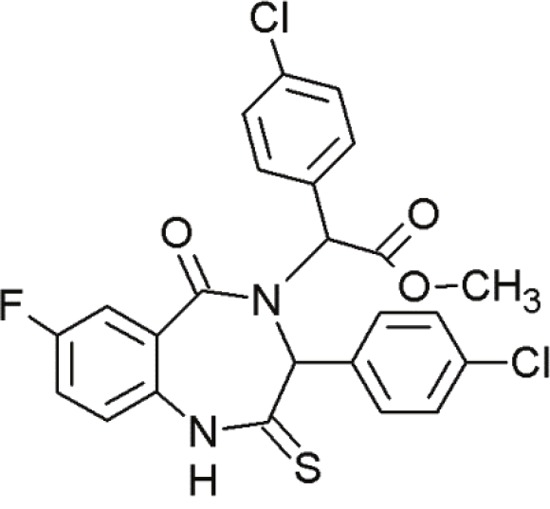	Benzo-diazepine-diones (BDPs)	Blocking the p53-binding pocket on MDM2 by mimicking p53	[117]

Although a large number of subsequently synthesized compounds achieved improved efficacies, they frequently exhibited poor pharmacokinetic properties and poor cell permeability. However, compound 27 demonstrated promising activity in MCF7 breast cancer cells [122]. *In vivo* studies with melanoma tumors showed that compound 27 enhanced the activity of doxorubicin, if applied in combination [123]. These compounds in combination, led to tumor growth inhibition at doses that are inactive, if administered alone. The combination resulted in reduced toxicity due to lower required dosages [83]. This points to the therapeutic benefit of small molecules in combination therapies.

Furthermore, several other thio-benzodiazepine compounds have been examined with respect to their structure-activity relationship and antitumor activity [95]. Several substances achieved high binding affinities with MDM2 and two of them, 8i and 8n, stood out particularly. These compounds showed excellent binding activities, which were even superior to the reference compound nutlin-3a [95]. Molecular docking studies of the thio-benzodiazepine-MDM2 complex illustrated that the binding interaction was mediated by three hydrophobic pockets that are filled by the three aromatic rings of the thiobenzodiazepines. The well positioned ester-group of the thio-benzodiazepines, functioning as hydrogen bond acceptor with Gly16, may account for the enhanced binding affinity [95]. Even *in vitro*, most compounds showed moderate to excellent cytotoxicity towards cancer cells. Some compounds, including 8i and 8n, showed even better biological activity than nutlin-3a in wt-p53 osteosarcoma cells [117]. Thus, compounds 8i and 8n represent promising new MDM2 inhibitors that require further evaluation.

#### Spiro-oxindoles

Yet another group of small molecules that hold large promise in inhibiting the p53-MDM2 interaction are the spiro-oxindoles [124] (Table 4, Table 7). The oxindole ring of these compounds mimics the side chain of Trp23 of p53, which is critical for MDM2 binding. The hydrophobic-substituted spiropyrrolidine-ring imitates the side chains of Phe19 and Leu26 [90, 124]. Extensive crystal-structure analyses have revealed that Leu22 is also critical for binding p53 and MDM2 [124], indicating the potential of oxindole derivates [92].

**Table 7:** Further, less relevant compounds of the MI-series

Agent	Chemical Structure	Agent Class	Mechanism of Action	Reference
MI-43	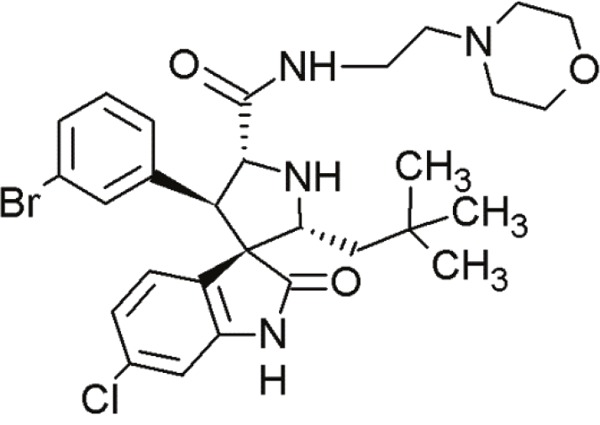	Spiro-oxindoles (MI-series)	Blocking the p53-binding pocket on MDM2 by mimicking p53	[126]
MI-63	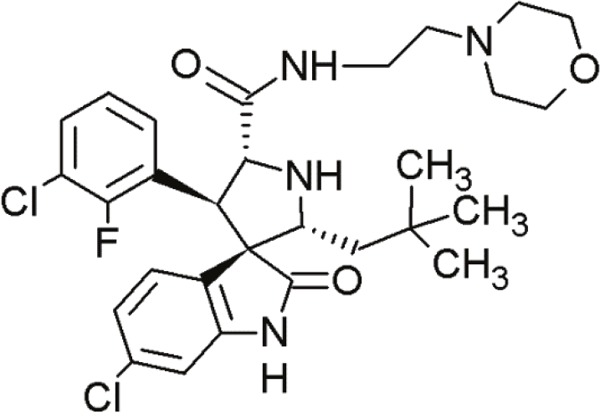	Spiro-oxindoles (MI-series)	Blocking the p53-binding pocket on MDM2 by mimicking p53	[83]

MI-63 and MI-219 are two spiro-oxindoles with improved structures. Although MI-63 showed prominent *in vitro* activity, its *in vivo* pharmacokinetic profile was unsatisfactory [92]. MI-219 on the other hand, was more effective owing to its high binding affinity for MDM2 and good oral bioavailability and better pharmacokinetic profile [92].

In a recent study by Samudio et al., the potential of MI-63 in acute myleoid leukemia (AML) revealed significance [125]. In AML cell lines and tissue samples, MI-63 elicited p53-dependent cytotoxicity as well as apoptosis. The disruption of MDM2-p53 interaction by MI-63 led to enhanced p53-levels and levels of target genes (e.g. p21). This also correlated with G1-cell cycle arrest and apoptosis induction [125]. Interestingly, MI-63 treatment depleted MDM4 (mouse double minute 4, also known as MDMX) protein levels [125]. MDM4, like MDM2, is an inhibitor of p53 activity [125]. The decline in MDM4-protein levels was proposed as consequence of MDM2-mediated proteasomal degradation [125]. Another plausible mechanism might be reduced transcription of MDM4 [125].

The drug-induced impediment of the MDM2-p53 interaction increased p53 levels, leading to the upregulation of p53 target genes and wt-p53-dependent induction of apoptosis in several human breast, colon and prostate cancer cell lines [105]. *In vivo* xenograft tumor experiments revealed a complete inhibition of tumor growth at non-toxic doses. Administration of MI-219 (300 mg/kg) for 14 days caused complete suppression of tumor growth, decreased the tumor volumes and showed no signs of toxicity [92, 98].

In another study by Sosin and colleagues, differential effects of MI-219 were tested against lymphoma cell lines and patient-derived non-Hodgkin's SLL (small lymphocytic lymphoma)/CLL (chronic lymphocytic leukemia) samples [91]. Compared to nutlin-3, MI-219 triggered an earlier response after treatment (nutlin-3: 48 h, MI-219: 12-24 h) and enhanced MDM2-autoubiquitination as well as degradation at equivalent concentrations [91]. Cell death induction by MI-219 was more effective and occurred earlier than by nutlin-3 [91]. Also, the enhanced efficacy of MI-219 was associated with a significant increase of p53-induced p53AIP1 (p53-regulated apoptosis-inducing protein 1) [91]. Reasons for MI-219s’ enhanced efficacy over nutlin-3 include differential binding affinities (7-fold higher) or variations in bioavailability [91]. Another reason for the increased efficacy of MI-219 is that MI-219 mimics four key binding residues in p53 (Phe19, Leu22, Trp23 and Leu26) in comparison to the three residues in the case of nutlin-3 (Phe19, Trp23 and Leu26) [91]. As a result, the former compound was subjected to chemical optimization in hydrogen binding and hydrophobic interactions [91].

One more compound amongst the arsenal of the MI-series, MI-43 preferentially inhibited the growth of wt-p53 cells. It induced growth arrest in G1 and G2 phases of cell cycle at low concentrations as a result of p21-induction. At higher doses, it induced PUMA/NOXA-evoked apoptosis. MI-43 was less toxic towards normal lung cells than cancer cells and sensitized cancer cells to etoposide-induced apoptosis, if used in combination [98] [105].

MI-888 is another new derivate with an excellent pharmacokinetic profile and enhanced *in vivo* efficacy [99]. It achieved a promising K_i_-value and produced rapid, complete and durable tumor regressions after oral administration in two human xenograft tumor models (osteosarcoma and acute lymphoblastic leukemia) [99].

SAR405838, also termed MI-77301, is another small molecule inhibitor of the p53-MDM2 interaction [93]. SAR405838 is a highly optimized compound in the spiro-oxindole family [127]. Its binding mode is similar to that of other spiro-oxindoles. It mimics the three key residues of p53 (Phe19, Leu26, Trp23) [93]. But unlike the other compounds, SAR405838 captures additional interactions. The C1 atom of its oxindole group shows hydrophobic interactions with MDM2. The imidazole side chain of His96 in MDM2 and the carboxyl group of SAR405838 interact *via* a hydrogen bond. Even π-π-stacking is present between the His96 of MDM2 and the 2-fluoro-3-chlorophenyl of SAR405838 [93]. In contrast with the co-crystal structures of p53-MDM2 and nutlin-MDM2, in SAR405838-MDM2, the N-terminus of MDM2 forms extensive interactions with SAR405838. This is due to re-folding of residues 10-18 of MDM2, which interact with SAR405838 through Val14 and Thr16 [93]. Through these additional interactions, SAR405838 achieves high binding affinities [93].

Bill et al. reported the preclinical effects of SAR405838 in both *in vitro* and *in vivo* dedifferentiated liposarcoma models [127]. SAR405838 restored downstream signaling through pharmacological MDM2 inhibition. The increase of p53-induced apoptotic genes *BAX* and *PUMA* suggests that the treatment activated the p53 pathway [127]. Expression of apoptosis-associated genes (*e.g*. p21, PUMA and BAX) was proportionate with increasing concentrations of SAR405838 [127]. This compound further demonstrated a promising oral pharmacokinetic profile in mice. Even if administered as single agent, it induced apoptosis at low concentrations [127]. SAR405838 promoted complete tumor regression after oral administration of 200 mg/kg/wk, whereas other potent MDM2-inhibitors (RG7112 and RG7388) did not achieve this effect [127]. The functional outcomes of SAR405838 strongly depend on the presence of MDM2 amplifications. Bill et al. showed that no effects were visible in wt-p53 liposarcoma cells without overexpression of MDM2. Therefore, SAR405838 treatment only induced apoptosis in cancer cells harboring *MDM2* amplifications [127]. In conclusion, SAR405838 showed very promising preclinical data. It is a potent and highly effective MDM2-inhibitor and is currently undergoing clinical trial [93].

#### RITA

RITA (reactivation of p53 and induction of tumor cell apoptosis) is a furan derivative [2,5-bis(5-hydroxymethyl-2-thienyl)furan] and was identified in a cell-based screen for wt-p53-reactivating molecules [20, 98] (Table 4).

RITA bound to the N-terminus of p53 (residues 1-63) and induced a conformational change, which propagated from the N-terminus to the core and C-terminal domain, advocating the interruption of p53 and MDM2 [12, 94, 98]. This led to p53 accumulation and induction of p53-dependent apoptosis in a variety of tumor cell lines of different origin including carcinomas of lung, colon, breast, skin; melanoma and diverse lymphomas [128]. Possibly, the binding of RITA to the N-terminus affected the hydrogen bonds within the MDM2-binding site, preventing the formation of the α-helix, which is necessary for MDM2 binding [20]. Low concentrations of RITA inhibited growth of carcinoma cells harboring wt p53, but showed minimal effect on cells lacking it [92]. In contrast to other MDM2 inhibitors, RITA activated p53 by binding to it instead of binding to MDM2 [92]. Furthermore, RITA suppressed the growth of human xenograft tumors in mice without causing toxic effects in normal tissues [68]. This led to the assumption that RITA may selectively activate p53 in tumor cells. Although nutlin-3a and RITA target the same protein complex, they induce different biological outcomes. Disruption of the p53/MDM2 complex may not be the only effect of these compounds [129]. Nutlin-3a changes the repertoire of MDM2-binding partners, whereas RITA affects p53 interactions. Burmakin et al. found that besides MDM2-p53 interaction, RITA also disrupted other p53 binding interactions such as with iASPP (inhibitor of apoptosis-stimulating protein of p53), Parc (p53-associated Parkin-like cytoplasmic protein) or E6-AP (E6-associated protein) [94]. In addition, p53 activated by RITA induced the expression of Fbxw7 (F-Box and WD repeat domain containing 7), which has a critical function in the degradation of N-Myc, a protein that correlates with a poor prognosis and resistance to therapy [94]. Transcription of Aurora A, an antagonist of Fbwx7-mediated degradation of N-Myc, was repressed by p53 [94]. In addition to its potential as wt p53 reactivating substance, RITA also exhibited paramount efficacy as mut-p53 activator.

The discovery of the mutant reactivating ability of RITA was serendipitous. Initially, Zhao et al. screened for wt-p53-activating substances [22]. RITA led to restoration of the p53-mediated transcriptional program and induction of p53-dependent apoptosis [68]. Remarkably, RITA reactivated a broad range of different p53 mutant species, including those that were mutated at three residues [83]. Therefore, a general mechanism of action has been suggested through which restoring the function of different types of mutants was achieved. However, the exact molecular mechanism has not been deciphered. RITA-induced cell death involved DNA-fragmentation, cytochrome C release, caspase activation, and apoptosis [83]. Zhao et al. found RITA to be more efficacious than PRIMA-1 in inducing cell death in a mut-p53-dependent manner. RITA achieved much lower IC_50_ values than PRIMA-1 [22]. Interestingly, the kinetics of cell death induction considerably differed between different cell lines. While death induction in skin cancer cells harboring mut-p53 protein took only 6-8 h, breast cancer cells required several days [22]. Such kinetic differences could result from differential kinetics of cellular uptake and/or degradation of RITA or a different set of p53 inhibitors/activators present in these cells [22].

Weilbacher et al. demonstrated that there was no strict link between cancer cell sensitivity to RITA and the p53 status in tumor cells. Even in p53-null cells, RITA was capable of inducing apoptosis [130]. Besides the fact that RITA interacts with TrxR1 (thioreduxin reductase 1) thus inducing ROS, it also promoted DNA damage [130]. Further, the authors proposed that JNK (c-Jun N-terminal kinases)/SAPK (stress-activated protein kinases) and p38 MAPK (mitogen-activated protein kinases) pathways are important in p53-deficient cells for caspase-dependent mitochondrial cell death [130]. JNKs translocate to the mitochondria, where, by modulating pro- and anti-apoptotic BCL-2 (B-cell lymphoma 2) family proteins, they activate BAX and BAK to initiate apoptosis [130].

The benefits of using compounds that target wt-p53 as well as mut-p53, like RITA, have been illustrated by Burmakin et al. They demonstrated that RITA restored wt- and mut-p53 activities and induced p53-dependent apoptosis in neuroblastoma *in vivo* and *in vitro* [94]. RITA treatment disrupted MDM2/p53-complexation and inhibited the interaction between MDMX (another regulator of p53) and p53 [94]. This results is of enormous importance, particularly in developing compounds that simultaneously target wt- and mut-p53 cancer cells. Such a treatment may reduce the emergence of drug resistance and enhance the clinical treatment successes [94]. Aziz et al. demonstrated that cancer cells can develop resistance to nutlin-3a [131]. Continuous treatment of mut-p53 cancer cells with nutlin-3a resulted in acquired p53 mutations [131]. These acquired p53 mutations could be a result of mis-repaired DNA breaks in cells that initiated, but did not finish the process of apoptosis [131]. Therefore, the development of therapies that simultaneously restore wt- and mut-p53 could be highly beneficial [94].

### Novel drug developments

#### Xanthone derivatives

**Figure 3:**
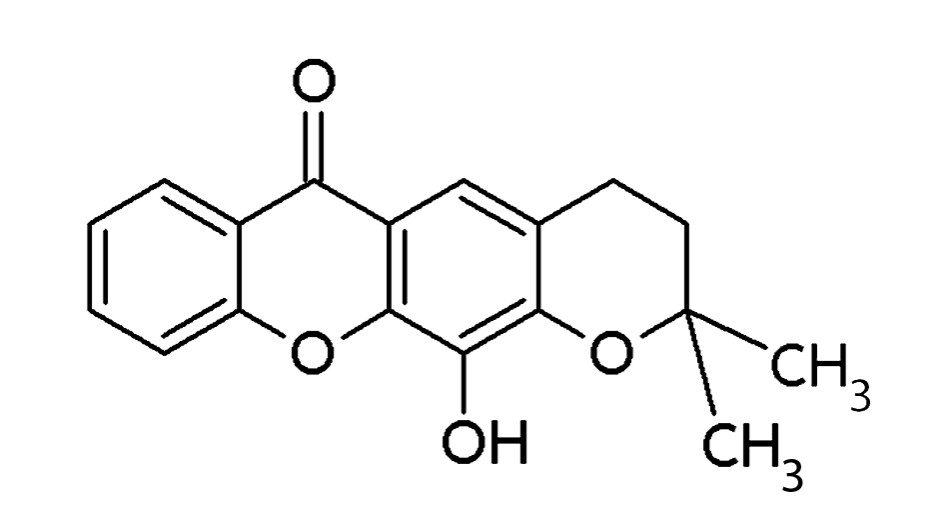
Structure of pyroxanthone 1

Xanthone derivates exhibit antitumor activity [132, 133]. Especially, prenylated xanthone derivatives have great potentials against breast cancer cell lines (MCF-7) harboring wt-p53 and overexpressed MDM2 [132]. Leão and co-workers identified a set of 14 putative MDM2 ligands with xanthone scaffold by virtual drug screening leading to the identification of pyroxanthone 1 (3,4-dihydro-12-hydroxy-2,2-dimethyl-2H,6H-pyrano [3,2-b]xanthen-6-on) [16] (Figure 3). This pyroxanthone derivative was effective against human tumor cells with wt-p53. It mimicked p53 activators by activating p53-dependent transcription and upregulation of p53 downstream signals. Pyroxanthone 1 bound to MDM2 in similar manner as nutlin-3a [16]. However, the compounds interacted differently. While the interaction between pyroxanthone 1 and MDM2 involved a hydrogen bond with Gly58, the nutlin-3a-MDM2 interaction mainly involved hydrophobic interactions [16]. Pyroxanthone 1 represented a useful lead compound for further structure-based design of more potent analogs.

#### Trisubstituted aminothiophenes

Another series of inhibitors of the p53-MDM2 interaction are derivatives of a novel scaffold (MCL0527) [27] (Table 8). These derivatives revealed commendable MDM2 binding affinities and anti-proliferative effects against several cancer cell lines [105]. Derivatization at the 2-amino group and modification at the 3-carboxy-group have been carried out to find more potent inhibitors [32]. Compound 24, showed the highest binding affinity effectively inhibited MDM2 binding, it did not exhibit sufficient cytotoxicity towards tumor cells. However, compound 9 inhibited both p53-MDM2 binding and tumor cell proliferation [32].

**Table 8:** Structures of 3,4,5-trisubstituted aminothiophenes

MCL0527	Compound 24	Compound 9
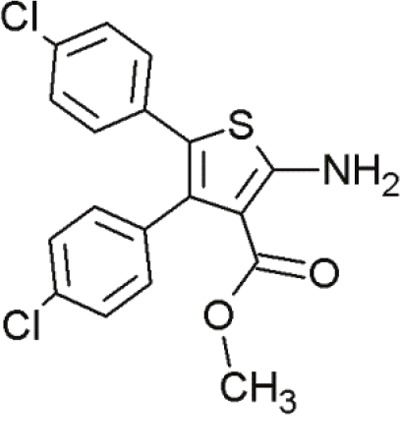	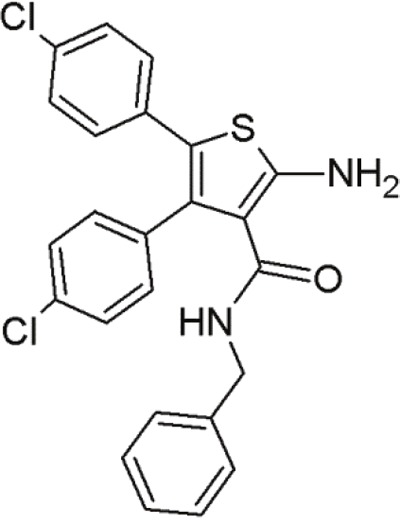	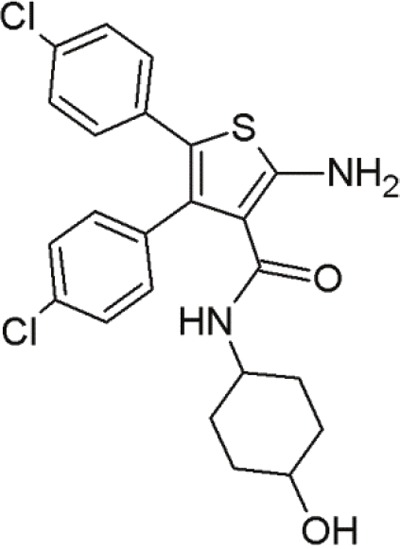

To examine possible binding modes of MCL0527 and compound 24, Wang and co-workers performed molecular docking studies [32]. The thiophene ring, an important core structure, revealed three hydrophobic substituents in MDM2 binding clefts. The two 4-chlorophenyl groups and one methyl ester/N-benzyl group mimicked the three key residues Leu26, Phe19 and Trp23 of p53 to form hydrophobic interactions. The methyl ester of MCL0527 did not occupy the spare room at the Phe19 binding site, as did the N-benzyl group of compound 24 [32]. This may be an explanation for its enhanced affinity. Some of these compounds exhibited even better anti-proliferative activity against wt-p53 cells than nutlin-3 [32]. Regarding wt-p53 selectivity, several compounds showed at least 3-fold inhibitory selectivity in wt-p53 cell lines compared to p53-null cell lines [32]. Hence, 3,4,5-aminothiophenes may be valuable contenders in cancer therapeutics targeting the MDM2-p53 interaction.

### Natural products as MDM2 inhibitors

#### α-Mangostin and gambogic acid

α-Mangostin and gambogic acid are prenylated xanthones derived from the mangosteen fruit of *Garcinia mangostana L. (Clusiaceae)* and resin of *Garcinia hanburyi* Hook.f. *(Clusiaceae)*,respectively (Table 9). Both α-mangostin and gambogic acid inhibited the p53-MDM2 interaction by binding to MDM2 [134]. Their cytotoxic activity against human cell lines [135, 136] as well as their antitumor activity in animals [137, 138] are already known. Gambogic acid induced apoptosis and cell cycle arrest in human tumor cells harboring wt-p53 [139]. Furthermore, α-mangostin showed cytotoxicity in human mut-p53 tumor cells [140]. p53-dependent transcriptional activity increased under treatment with these compounds, and thereby the negative effect of MDM2 on p53 was inhibited [134]. The predicted binding interactions between α-mangostin/gambogic acid and MDM2 are similar to those between nutlin-3a and MDM2. These compounds bind near the p53-binding site, filling the space that is necessary for the interaction with α-helix motifs in the p53 amino terminal domain. The two compounds revealed high binding affinities with the residues Gly58, Asp68, Val75 and Cys77 of the hydrophobic MDM2 binding site. Only gambogic acid formed hydrogen bonds with residues Gln72 and Phe55 of MDM2 [134, 141].

**Table 9:** Naturally derived compounds α-mangostin and gambogic acid

α-Mangostin	Gambogic acid
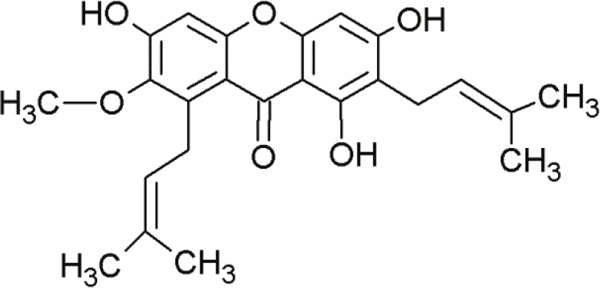	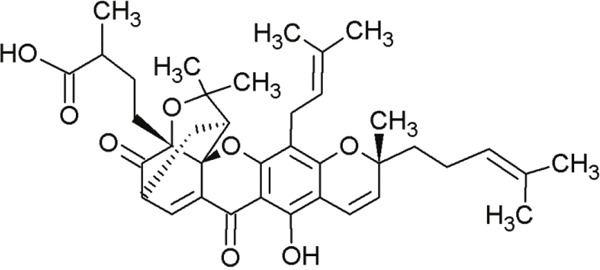

#### Siladenoserinols

**Figure 4:**
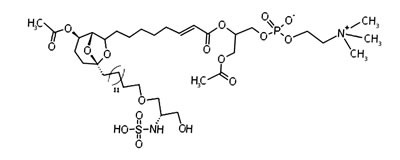
Structure of the marine compound siladenoserinol A

The screening of extracts from marine invertebrates in quest of MDM2 inhibitors led to the identification of 12 sulfonated serinol derivatives (siladenoserinol A-L) from tunicates belonging to the Didemnidae family [142]. Each of them contained a 6,8-dioxabicyclo [3.2.1]octane unit with either glycerophosphocholine or glycerophosphoethanolamine moieties and inhibited the p53-MDM2 interaction [142]. Despite their structural similarities, they showed extreme differences in their IC_50_ values. siladenoserinol A emerged as the most cytotoxic compound [142] (Figure 4). More studies are warranted to optimize the inhibitory effect of siladenoserinols and elucidate their molecular mechanisms.

## SMALL MOLECULES: NEW OPPORTUNITIES WITH CHALLENGES

In the recent years, great efforts have been invested in the evaluation of novel small molecules that could act on the MDM2-p53 axis and function as mut-p53 reactivators. Despite initially promising outcomes only a few of the discovered small molecules exhibited the right combination of suitable properties to justify their entry into clinical trials. Many of the compounds were characterized by poor pharmacokinetic profiles.

RG7112, an advanced compound of the class of cis-imidazolines with high efficacy and selectivity, is currently in clinical trials [89]. However, serious side effects of RG7112 posed a critical issue for its clinical use [143]. Besides gastrointestinal toxicity (including nausea and vomiting), hematological toxicity (neutropenia and thrombocytopenia) was also observed [143–145]. Therefore, strict clinical monitoring is required and the effects of long-term exposure need to be evaluated [143].

A major obstacle to the clinical use of wt-p53 activators is the acquisition of p53 mutations during treatment [146]. Maki and coworkers demonstrated that wt-p53-harboring cancers lead to resistant clones with acquired p53 mutations, if chronically treated with nutlin-3a [131]. At this juncture, it must be kept in mind that overexpression of MDM4, a MDM2 homolog and another potent negative regulator of p53, also led to decreased efficacy of nutlin-induced p53-activity and caused inadequate responses to nutlin treatment [147].

It is also possible that activation of p53 may lead to senescence, but not apoptosis, which could be hazardous in long term use [146]. Mirzayans et al. supported this assertion that the primary response to p53 activation in wt-p53 cancer cells may represent a form of senescence [148]. This may be problematic, if these cells eventually escape senescence and re-enter cell cycle [146].

A further challenge that needs to be dealt with is the potential of activating wt-p53 in non-cancerous, healthy tissues, which could have toxic effects [146]. This effect has been studied using mice with a hypomorphic allele of MDM2. These mice showed a phenotype with increased p53-dependent apoptosis in lymphocytes and epithelial cells [149]. This did not affect the lifespan of the mice, but it did affect their size [146]. Thus, it remains to be seen whether the use of MDM2-antagonists in clinical trials may exhibit similar effects. This concern enhances the need for highly specific agents.

Another critical factor to consider is the limited efficacy of p53-based cancer therapies in cells lacking the ability to phosphorylate p53Ser46, which subsequently increases the affinity of p53 to pro-apoptotic genes [150]. Ma et al. determined that neither nutlin-3, nor RITA were able to induce p53-mediated apoptosis in cancer cells that are unable to phosphorylate p53Ser46 [150]. Therefore, dysregulation of phosphorylation in those cancer cells might be a predicting factor of failed response to therapy.

## SMALL MOLECULES IN CLINICAL DEVELOPMENT

Although some mut-p53 activators as well as MDM2-antagonists have shown potent activity *in vitro*, only a few compounds demonstrated desirable pharmacokinetic properties and tolerable toxicity profiles *in vivo*. Thus, the number of compounds in clinical trials remains relatively few. Among the best known inhibitors are cis-imidazolines, such as nutlins and spirooxindoles [16]. Table 10 gives an overview of some important small molecules targeting the p53 pathway that have entered clinical trials.

**Table 10:** Some important p53 activators in clinical trials [https://clinicaltrials.gov/]

Compound	Mechanism of action	Status	ClinicalTrials.gov identifiers
**PRIMA-1**^Met^ **(APR-246)**	Small molecule; mut-p53 reactivator	Phase I in hematological an prostatic neoplasms (completed)	NCT00900614
**PRIMA-1**^Met^ **(APR-246)**	Small molecule; mut-p53 reactivator	Phase Ib/II in ovarian cancer with carboplatin/PLD (recruiting)	NCT02098343
**RO5045337 (RG7112)**	Small molecule; MDM2 antagonist	Phase I in advanced solid tumours, solid tumours, haematological neoplasms and liposarcomas (all completed)	NCT00559533 NCT01164033 NCT00623870 NCT01143740
**RO5045337 (RG7112)**	Small molecule; MDM2 antagonist	Phase I in AML with cytarabine (completed)	NCT01635296
**RO5045337 (RG7112)**	Small molecule; MDM2 antagonist	Phase I in soft tissue sarcoma with doxorubicin (completed)	NCT01605526
**RO5503781** **(RG7388)**	Small molecule; MDM2 antagonist	Phase I in AML as single agent or with cytarabine (active, not recruiting)	NCT01773408
**RO5503781** **(RG7388)**	Small molecule; MDM2 antagonist	Phase I in advanced malignancies except leukemia (completed)	NCT01462175
**SAR405838 (MI-77301)**	Small molecule; MDM2 antagonist	Phase I in malignant neoplasms (active, not recruiting)	NCT01636479
**SAR405838 (MI-77301)**	Small molecule; MDM2 antagonist	Phase I in malignant neoplasms with pimasertib (recruiting)	NCT01985191

PLD, Pegylated Liposomal Doxorubicin Hydrochloride

AML, Acute Myelogenous Leukemia

## CONCLUSIONS

Cancer is a complex conglomeration of diseases. Elucidating the mechanisms of cancer remains elusive. This necessitates the search for novel strategies to overcome the challenge. Mutated p53 is not only a key player in carcinogenesis, but is also associated with resistance to established cytotoxic anticancer drugs such as cisplatin, epirubicin, 5-fluorouracil, methotrexate and many other chemotherapeutics. However, contradictory reports pertaining to different aspects of p53's actions prevent from devising fool-proof intervention strategies. For example, while some studies confirmed strong connections between p53 mutations and drug resistance, others did not. Methodological differences may at least in part account for the diverging results in the literature. Several reasons may contribute to these discrepancies, *e.g*. non-standardized methods for p53 status evaluation, differences concerning patient selection, different polychemotherapy regimens, duration of follow-up, *etc*. Thus, the general prognostic status and role of p53, though mainly positively confirmed, remains controversial to some extent. Due to the high frequency of p53 mutations in human tumors, this tumor suppressor is an important target for novel anticancer therapies. Several research teams have dealt with the possibilities of restoring p53's function to treat cancer and their efforts showed worthwhile outcomes. Many novel molecules have been identified so far to restore p53's wild-type conformation and thereby recover its tumor suppressive function. The results were also promising regarding the combination of small molecules with conventional anticancer drugs. For example, synergistic effects between PRIMA-1/APR-246 and cisplatin have been shown *in vivo* and *in vitro*. However, further studies are required to develop specific small molecules with good pharmacokinetic profiles and acceptable tolerability in patients. Despite the fact that p53 mutations promote tumorigenesis, other mechanisms have also to be kept in mind. Among mechanisms concerning modifications in the pro- and antiapoptotic balance, even mechanisms involving drug uptake or export, changes molecular targets or DNA repair as well as mechanisms concerning the metabolic prodrug activation or drug inactivation are possible mechanisms causing drug resistance [151]. The fact that one half of all cancers express wt-p53 suggests the importance of investigating other members of the p53 pathway. Recently, small molecules capable of switching off the activity of MDM2, is a negative regulator of p53, have been identified. Novel MDM2 inhibitors increased the activity of combination treatment with standard chemotherapy. However, only few compounds possess desirable pharmacokinetic properties and acceptable toxicity profiles and further investigations are urgently needed.

In the future, combination therapies consisting of standard cytotoxic drugs and novel small molecules targeting p53 and MDM2 may be the key to fight cancer. Overcoming resistance to classical anticancer drugs by exploitation of synergistic effects of novel small molecules bears a huge potential to substantially improve the outcome of cancer chemotherapy. This goal is certainly not a trivial task, but is worth doing for the sake of alleviating the devastating consequences of the disease.
